# Obstetric interventions’ effects on the birthing experience

**DOI:** 10.1186/s12884-024-06626-5

**Published:** 2024-07-27

**Authors:** Anna Volkert, Lisa Bach, Carsten Hagenbeck, Jan Kössendrup, Charlotte Oberröhrmann, Mi-Ran Okumu, Nadine Scholten

**Affiliations:** 1 Chair of Health Services Research, Institute of Medical Sociology, Health Services Research and Rehabilitation Science, Faculty of Medicine and University Hospital Cologne, University of Cologne, Cologne, Germany; 2 Department of Gynecology and Obstetrics, Medical Faculty and University Hospital Düsseldorf, Heinrich Heine University Düsseldorf, Düsseldorf, Germany; 3 Center for Health Communication and Health Services Research, Department for Psychosomatic Medicine and Psychotherapy, Faculty of Medicine, University Hospital Bonn, Bonn, Germany

**Keywords:** Birth experience, Birth interventions, Episiotomy, Fundal pressure, Instrumental delivery, Cesarean section

## Abstract

**Background:**

The birth experience plays a pivotal role in the mother´s mental well-being and has a crucial effect on the mother-child bond. Unanticipated medical interventions, including fundal pressure, episiotomy, assisted vaginal delivery (AVD), or unplanned cesarean section (CS) during labor, may adversely affect the birth experience. The objective of this study is to identify factors contributing to the diminished evaluation of the birth experience after assessing the prevalence of unplanned obstetrical interventions in Germany.

**Methods:**

For this cross-sectional analysis, 4000 mothers whose children were born 8 or 12 months before were asked about their birth experience via a paper-based questionnaire. Overall 1102 mothers participated in the study, representing a response rate of 27.6%. The revised Childbirth Experience Questionnaire (CEQ2) was used to measure the childbirth experience. In addition to descriptive and bivariate analyses using the Wilcoxon rank-sum test and Kruskal-Wallis-test, we calculated multivariate linear regression models for each dimension of the CEQ2.

**Results:**

In general, the participants evaluated their childbirth experience favorably, assigning an average rating of 3.09 on a scale ranging from 1 to 4. Women who experienced fundal pressure, an AVD, or an unplanned CS rated their birth experience significantly worse compared to women who gave birth without interventions. Unplanned CSs received the lowest ratings for “personal capability” and “perceived safety,” and an AVD resulted in lower scores for “professional support” and “participation.” However, the interventions we studied did not account for much of the variation in how the childbirth experience plays out for different individuals.

**Conclusion:**

Obstetric interventions have a significant effect on different dimensions of the birth experience. If a high level of birth satisfaction is to be achieved, it is important to know which dimension of satisfaction is affected by the intervention so that explicit measures, like fostering communication, participation or safety can be taken to promote improvement.

**German Clinical Trial Register:**

DRKS00029214, retrospectively registered (Registration Date 22.06.2023).

**Supplementary Information:**

The online version contains supplementary material available at 10.1186/s12884-024-06626-5.

## Introduction

The birth experience is a crucial factor that significantly impacts the development of postpartum stress disorders [1, 2], the mother-child bond [3, 4], the decision to have further children [5], and the choice of future birthing methods [6]. According to the FIGO statement on respectful maternity care, obstetric care should be supportive, individualized, and evidence-based [7]. The birth experience is significantly influenced by the self-efficacy experienced during birth, the possibility to actively participate in the decisions of the birth process and being well-informed and empowered [8–10]. This is also reflected in the fact that global birth satisfaction is made up of individual dimensions that can be measured and influenced individually [11].

Research has demonstrated that, obstetric interventions can influence the mother’s satisfaction with the birth experience, with fewer interventions generally leading to higher satisfaction levels [6, 12]. Obstetric interventions are often employed to improve maternal and/or fetal birth outcomes. To expedite delivery in cases of fetal distress, practitioners may utilize techniques such as fundal pressure [13], episiotomy [14], assisted vaginal delivery (AVD) using forceps or vacuum extraction (VE) [15], or unplanned cesarean section (CS) [16].

Fundal pressure, also known as the Kristeller maneuver, is applied by obstetricians/midwives to enhance the downward progression of the fetus through the birth canal by applying suprapubic pressure on the maternal abdomen [13]. A study from the US in 2005 found fundal pressure rates of 5% [17], and in Austria, fundal pressure was practiced in 23% of births [18]. While no data exist on the prevalence for Germany, the German guideline states that fundal pressure should be avoided if possible and, if done, only after strict indication [19]. From the mother’s perspective, pain during fundal pressure or a sense of powerlessness due to a lack of information or opportunities for participation has been reported [20, 21].

Although there is only weak evidence, an episiotomy can be performed to accelerate delivery in cases of suspected fetal hypoxia or to prevent obstetric anal sphincter injury in vaginal operative deliveries [22]. In Western countries, the episiotomy rate is below 30% on average and shows a downward trend, unlike in developing countries, which often still have episiotomy rates of over 70% [23]. Birthing mothers are often not informed about an episiotomy [24], and this could negatively influence the birth experience [25, 26].

Other medical procedures to expedite the birth of a potentially compromised fetus include AVD, and unplanned CS, whereby AVD seems preferred over unplanned CS from the mother´s perspective [27, 28]. The rate of AVD in Germany is around 7% [19], which is twice as high as in the US [27]. Studies indicate a negative association between AVD and birth satisfaction [29, 30]. Unplanned CS is another intervention that influences the birth experience not only by changing the mode of birth but also by possibly triggering a feeling of powerlessness and helplessness in the birthing person [31].

All the interventions referred to occur unplanned at a time when the health of the mother or fetus appears to be at risk or the birth process is protracted. At the same time performing a medical intervention shifts the physiological birth process to a medical procedure [32]. This vulnerable phase presents a particular challenge to caregivers in terms of providing respectful maternal care, as it is essential not only to ensure the safety of the mother and the fetus, but also to involve the mothers individually in medical decision-making and respect their personal needs [33].

Even though the influence of obstetric interventions on global satisfaction has already been investigated, there has not yet been a specific examination of the effects of obstetric interventions on the individual dimensions of satisfaction [34].

One approach to encompassing these aspects from the mothers’ perspective is through the utilization of the revised version of the Child Birth Experience Questionnaire (CEQ2), a validated survey instrument that explores four different dimensions of maternal satisfaction with the childbirth experience [35]. Knowledge of the impact of obstetric interventions on the individual dimensions of birth satisfaction, like perceived safety or participation is important for taking targeted countermeasures to ensure satisfaction with the birth experience.

The aim of this analysis is therefore to gain a better understanding of the association between unplanned medical interventions and the individual dimensions of maternal childbirth experiences (CEQ2).

## Methods

The cross-sectional data are derived from a questionnaire survey of mothers 8 and 12 months after birth. This survey was conducted in cooperation with two statutory health insurance companies as part of the MAM-Care study (FKZ 01GY2110).

From all insured persons meeting the inclusion criteria, the health insurers drew a random sample of 4000 mothers who were invited to participate in the study. The inclusion criterion was the documented birth of a live-born child (ICD codes Z37.0 “Singleton, live-born,” Z37.2 “Twins, both live-born,” or Z37.5 “Other multiples, all live-born”) in a German hospital in May or September 2022. Consent to the anonymous survey was given implicitly by returning the questionnaire. No personal data were collected and no information from the health insurance company was linked.

**Fig. 1:**
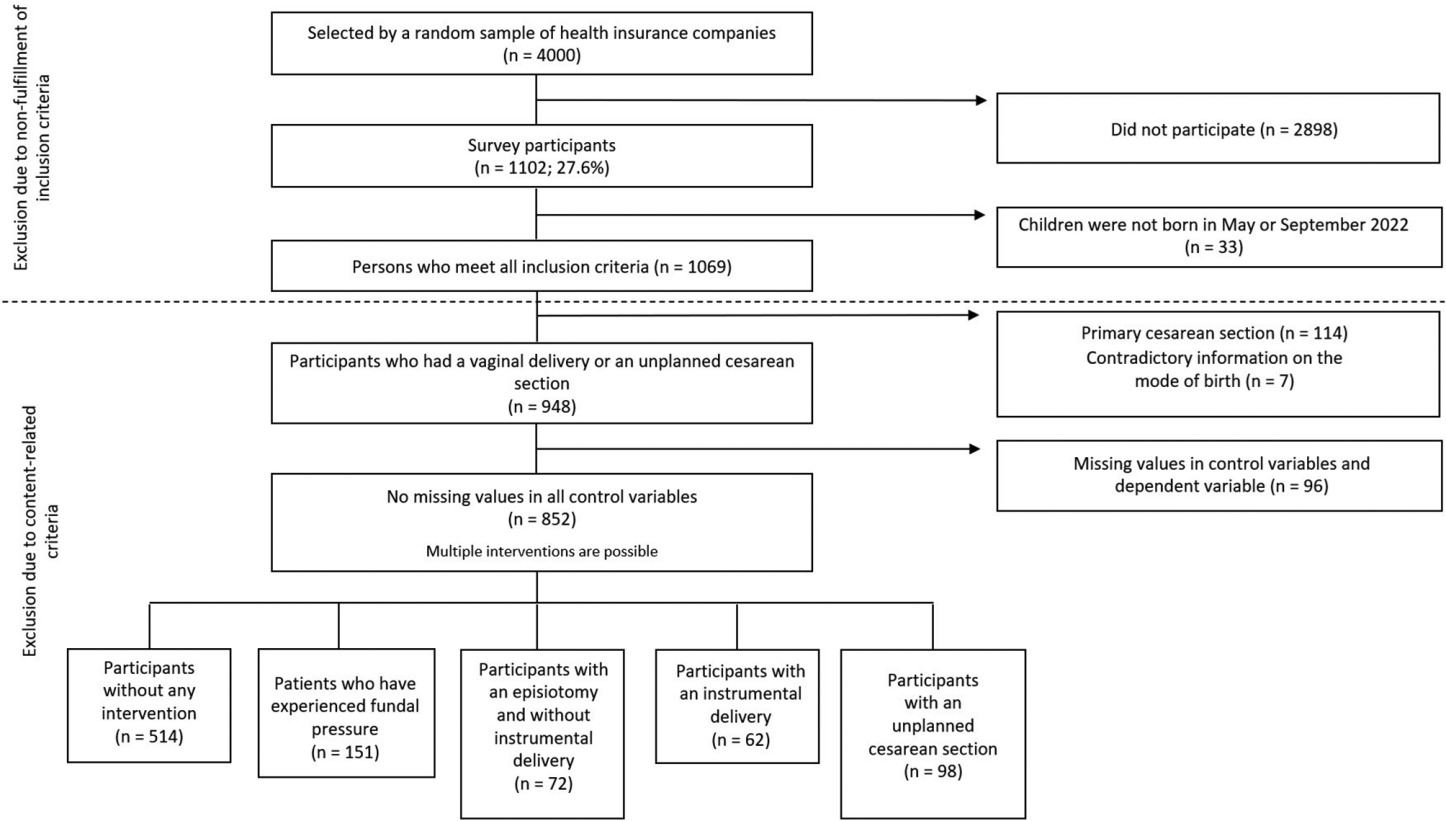
**Sample selection**

Overall 1102 mothers participated in the study, corresponding to a response rate of 27.6% (Fig. 1). Data from mothers who did not meet the inclusion criteria (birth in May or September 2022) were removed from the dataset. In addition, mothers with a planned CS and with contradictory information on the mode of birth were excluded from this analysis. In addition, 97 cases were removed due to missing values in the analysis variables. The data from two respondents who indicated their year of birth instead of their age were recoded.

### Dependent variable

We used the revised version of the Childbirth Experience Questionnaire (CEQ2), a validated survey instrument that explores various dimensions of maternal satisfaction with the childbirth experience [11, 35]. The instrument comprises four domains: “Own Capacity,” “Professional Support,” “Perceived Safety,” and “Participation.” It has undergone international validation and has been employed in various studies [35–37]. The “Own Capacity” domain serves to document the woman’s coping mechanisms during the birthing process, her perceived level of control, and the alignment between her expectations and the actual experience [35]. This aspect is addressed in CEQ2 through the participation dimension, which assesses the woman’s level of feeling informed, her involvement in decisions, and the consideration of her opinion [35]. Another decisive factor in the decision-making process is how supported a woman feels by medical staff. This is addressed by the “professional support” dimension and contains respectful maternity care, empathy and privacy [35]. The aspect of “perceived safety” focuses on the woman’s emotions and her sense of security throughout the childbirth process. We applied the CEQ2 to assess the birth experience from the birthing persons` perspective [35]. To enable the international comparability of our results, we used the validated German translation [38] with the item structure of the original scale [35]. Furthermore, to ensure that mothers who have had a cesarean section also feel addressed, we have adapted the wording in consultation with the authors of the German translation and replaced “during labor and delivery” with “during the birth process” (CEQ2.0-R). The response options for the items in all four domains ranged from 1 (totally disagree) to 4 (totally agree) on a four-point Likert scale. Items worded in reverse have been recoded accordingly. All four dimensions of the scale are considered individually in the descriptive presentation and in the analyses (Tables 2 and 3). The individual dimensions comprise the mean values of the associated items. To calculate the total score, the values of the individual dimensions sub scores were summed up and then divided by four.

### Independent variables

The medical interventions considered are fundal pressure, episiotomy, AVD (forceps and VE) and unplanned CS. There were yes and no response options to the questions “Was pressure applied externally to the abdomen by medical personnel during birth?” “Was an episiotomy performed during birth?“ and “How did you give birth: vaginal with forceps / vaginal with VE?“. The birth mode unplanned CS was assumed if CS was selected as the birth mode, and it was unplanned (i.e., occurred after the onset of labor or after induction).

In addition to the hypothesis-testing study variables, the following confounders were included in the analyses: maternal age, primipara vs. multipara, educational level (at least a university degree vs. Abitur [equivalent to at least 12 or 13 years of schooling [(High School)] vs. no Abitur), birth weight higher 3500 g and the child´s birth month (May vs. September 2022) to consider possible COVID-19 associated influences.

### Statistical analysis

To explore the association of medical interventions with the reported birth experience, we assessed the impact of interventions on the individual dimensions of the CEQ2 using both the bivariate Wilcoxon rank-sum test and the Kruskal-Wallis test. Subsequently, linear regression models with robust estimators were built for each dimension of the CEQ2, since the normal distribution assumption was not fulfilled (Shapiro-Wilk test) and heteroskedasticity could not be excluded (test of Szroeter was negative, Breusch-Pagan and Cook-Weisberg test was positive). The analyses were conducted using the software Stata 16.1 and R 4.2.0 with the *ggplot2*, *Gtsummary* and *jtools* packages [39–42].

## Results

The average age of the birthing person was 33.19 (SD 4.01). Table 1 shows the characteristics of the study population. The most frequently experienced intervention in the sample was fundal pressure (19%); a total of 12% of the respondents gave birth via an unplanned cesarean section, while 8% gave birth via instrumental birth. An episiotomy was performed on 14% of the respondents.

When interventions are examined based on individual dimensions (Fig. 3), it becomes apparent that women tend to rate their coping options lower for AVD and unplanned CS compared to those who experienced fundal pressure or an episiotomy (only women who have undergone an episiotomy without any other intervention are considered here). In all individual dimensions, the highest score was attained for vaginal birth without intervention. Regarding interventions, no consistent pattern was observed, with unplanned CS obtaining the lowest scores for “own capacity” and “perceived safety.” In contrast, for “professional support” and “participation,” AVD yielded lower scores than unplanned CS.

**Table 1:** **Characteristics of the study population**

Characteristic		*n* = 852*	Mean CEQ2	*p*-value^×^
Fundal pressure	No	625 (81%)	3.17	< 0.001
	Yes	151 (19%)	2.92	
	Unknown	76		
Episiotomy	No	642 (86%)	3.16	< 0.001
	Yes	104 (14%)	2.96	
	Unknown	106		
Assisted vaginal delivery	No	692 (92%)	3.17	< 0.001
	Yes	62 (8%)	2.77	
	Unknown	98		
Unplanned cesarean section	No	754 (88%)	3.14	< 0.001
	Yes	98 (12%)	2.73	
Parity	Nulliparous	417 (49%)	3.03	< 0.01
	Multiparous	435 (51%)	3.15	
Birth weight	< 3500 g	447 (52%)	3.05	0.12
	≥ 3500 g	405 (48%)	3.14	
Education	No Abitur	126 (15%)	3.12	0.38^+^
	Abitur	226 (27%)	3.11	
	University	500 (59%)	3.08	

*n(%); ×Wilcoxon rank sum; test + Kruskal–Wallis test. Own calculations

Table 2 shows the CEQ2 in its four dimensions. Overall, the person giving birth rated their birth experience as positive (mean 3.09 on a scale from 1 to 4), and only 5% of the respondents gave a CEQ2 score of 1.9 or below. A review of the individual dimensions reveals a high level of satisfaction with the professional support during birth and the opportunity to participate. The lowest rating was given to their coping skills (own capacity).

**Table 2:** **Dimensions of the CEQ2**

Dimensions	Mean	Median	SD*
Own capacity	2.59	2.63	0.63
Perceived safety	3.18	3.33	0.71
Professional support	3.31	3.40	0.68
Participation	3.30	3.33	0.72
Total score	**3.09**	**3.21**	**0.57**

*n* = 852. *Standard deviation. Own calculations

**Fig. 2:**
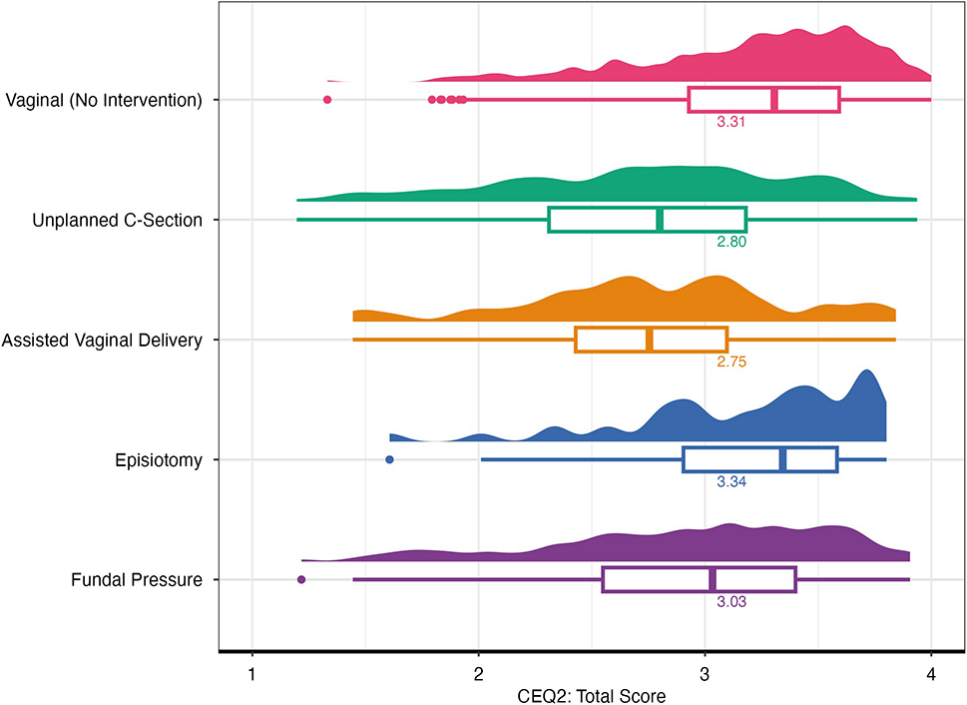
**Boxplots of the CEQ2-Total score by intervention**

Regarding the overall assessment, the greatest satisfaction, and therefore, the highest were given for vaginal births without medical intervention (Fig. 2). The birth experience was rated worst for AVD and unplanned CS.

**Fig. 3:**
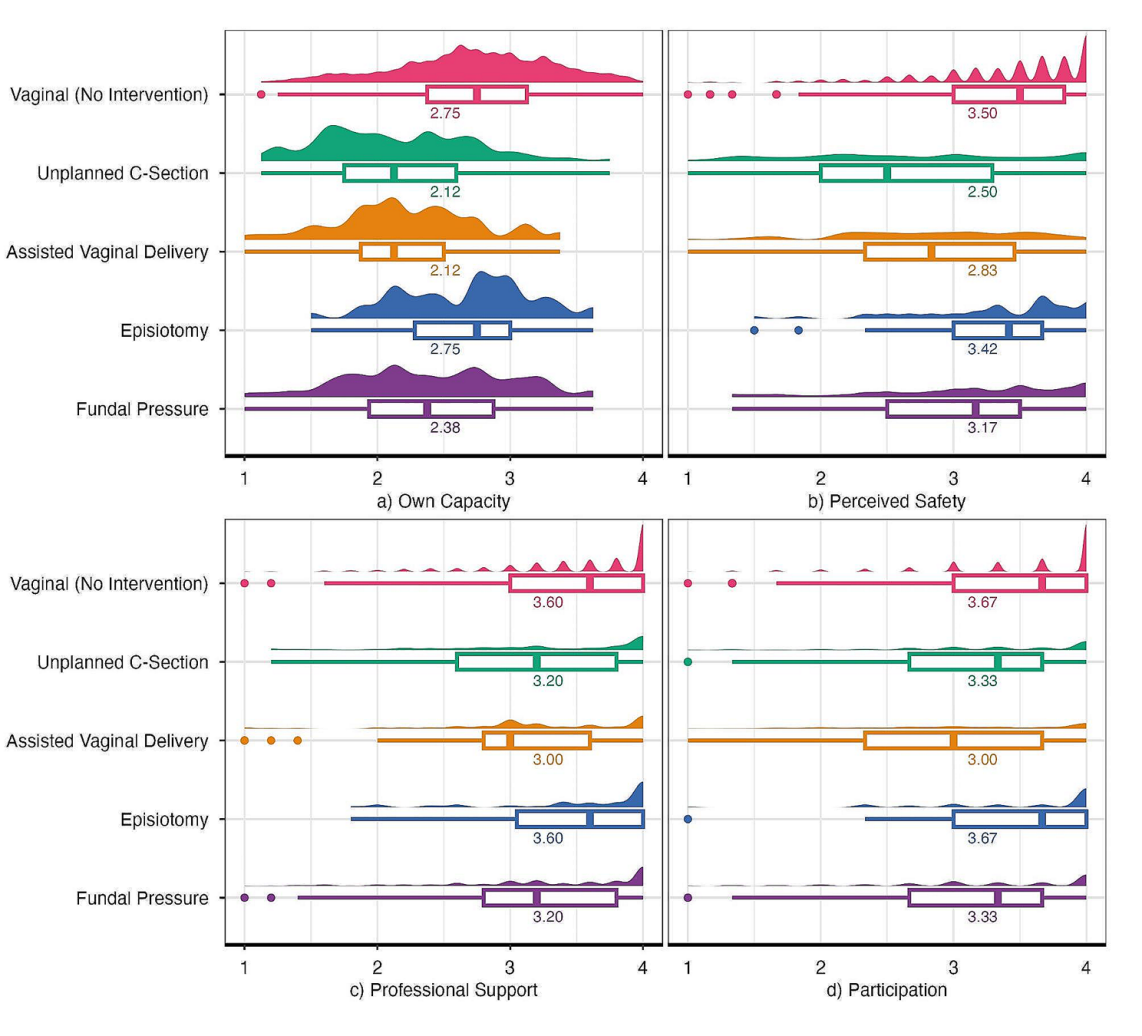
Boxplots of the individual subdimensions by intervention

For the multivariate analyses, an initial model was computed for each dimension (Table 3, Additional file 1). In all the models, the association of the intervention compared to vaginal birth without intervention on the individual dimensions of the birth experience is calculated, while considering confounders such as maternal age, education, parity, birth weight and birth period. Across all the models, there is a significant negative association with AVD and fundal pressure. A similar pattern is observed for unplanned CS, with significant associations in all dimensions except “participation”.

## Discussion

Our analyses aimed to compare obstetric interventions used in the second stage of labor and their effect on different dimensions of the birth experience.

On average, the respondents´ CEQ2 score was 3.09 on a scale from 1 to 4, with 5% of respondents having a CEQ2 score of 1.9 or less, and therefore, experiencing the birth of their child as very negative overall. Moreover, women who had none of the observed medical interventions rated both the entire birth and its sub-dimensions significantly better than women who had experienced one of the analyzed interventions.

Fundal pressure is known to have a negative association with the birth experience, and there has been ongoing controversy surrounding this issue. Although this intervention is internationally classified as potentially dangerous and ineffective and, according to the German guidelines, should not be exercised, if possible [19], it was performed in about one-fifth of births in our sample. Although the women who had experienced fundal pressure gave higher scores in all dimensions than women with AVD or CS, they rated all dimensions except professional support significantly worse than women who had no interventions. The reason for this may lie in the experienced self-efficacy, which tends to be perceived as less effective and lower in women who experienced fundal pressure [43]. Our results show that further efforts should be made to support womens´ self-efficacy (own capacity) during the birthing process. Based on the current evidence and the very critical voices that speak out against the use of fundal pressure [44], it can be questioned to what extent fundal pressure should still be applied at all, or at least efforts should be made to reduce the frequency of fundal pressure.

The impact of episiotomies on the individual dimensions of the CEQ2 is not evident in the multivariate analysis. From this perspective, it can be inferred that adequate support by the staff is already in place, or episiotomy may not be fundamentally relevant for the birth experience, as Bossano et al. pointed out [45]. However, it is important to note that the potential long-term effects of episiotomies are not considered in this analysis.

AVD and unplanned cesarean section have the strongest negative association with the CEQ2 score. This result is consistent with findings from other research using the CEQ2 [36, 46] and other research in this field [47, 48]. An examination of the individual dimensions reveals that women who had AVD rate their capacity and participation, in particular, as significantly worse than women who did not receive this intervention. Descriptive and bivariate analyses conducted by other researchers in similar studies have yielded comparable results [35, 36]. Therefore, the empowerment of women giving birth is particularly essential for a positive birth experience despite instrumental delivery [49]. However, cooperation and empowerment, in particular, interact with the medical staff providing care [49]. Compared to all other interventions, women who had an AVD rated both professional support and the assessment of having sufficient opportunities to participate during childbirth the lowest. This result is not consistent with the findings of a previous study that examined women with induced labor using the original CEQ, where caesarean section was rated as less favorable than AVD in all dimensions [37]. Although Germany´s AVD rate of 8% is lower than England´s [50], for example, data from the US (2.5%) show that a further reduction in the AVD rate is possible [51] whereby this may lead to an increase in the CS-rate.

Women who had an unplanned CS rated their overall birth experience slightly worse than women who had a AVD – even though there are only minimal differences between the two. This evaluation of the unplanned CS confirms findings from another study that report higher satisfaction ratings for instrumental birth when compared to emergency CS [47]. Based on the individual dimensions, the explanation for mothers’ bad experiences with CS lies particularly in perceived low self-efficacy (own capacity) and concerns about the safety of the baby and oneself (perceived safety). However, the moderating effect of the support experienced by the staff on satisfaction with the birth experience must always be considered [52]. Therefore, support from the staff can mitigate the negative effect of the cesarean birth mode. Our data show that women who had an unplanned CS (median 3.2) rated professional support better than women who had a AVD (median 3.0). Regarding participation, differences were observed between women who had experienced a AVD and those who had experienced a CS. From this result, it can be deduced that even in the case of CS, it was possible from the mothers’ point of view to participate and be part of decision-making processes. However, the differences regarding participation in vaginal birth are not significant in the multivariate model.

The results, particularly for AVD and CS, indicate that professional support, communication, empowerment and opportunities for participation are crucial for the woman giving birth. This particularly applies to the phase of childbirth, characterized by exhaustion and possibly concern for the health of the mother and/or fetus. This highlights the significance of raising awareness among obstetric personnel about the occurrence of traumatic procedures and equipping them with techniques to prevent adverse experiences and trauma. Overall, it is worth discussing why individual interventions per se play only a minor role in explaining the total birth experience. Our analysis demonstrates that negative experiences in one aspect of the CEQ2 can be compensated for by positive experiences in other areas. It is more meaningful to examine individual dimensions rather than the overall score. In practical terms, this implies that an intervention, does not necessarily result in a negative birth experience when the patient feels safe and is professionally supported by the staff. Furthermore, it is important to provide mothers with the opportunity to experience their own self-efficacy during interventions and to participate in the birth. This highlights that establishing contact, fostering involvement, providing support, and making efforts to alleviate anxiety can contribute to a positive birth experience despite interventions.

### Limitations

Overall, with 1102 questionnaires returned a moderate response rate of 27.6% was achieved. The survey documents were sent to the participants by post, ensuring that all information was provided in written form to the respondents. This means that the distribution of the questionnaires did not depend on the clinical staff and all participants receive the same information, thus avoiding a selection bias trough in the maternity clinic. At the same time, however, this also means that no information about the maternity clinic is available and there is no additional possibility to motivate women to participate.

The two cooperating health insurance funds cover around 17.5% of people with statutory health insurance in Germany. The health insurance companies are one nationwide and one regional health insurance company, which only covers North Rhine and Hamburg. Compared to the population as a whole, only a few mothers with a low education level and a migration background were reached. It is possible that these vulnerable groups have more negative birth experiences than those with a higher education level and/or are native Germans.

A further limitation of the study is that the interventions included in the multivariate models only account for a small proportion of the variance. This is a hint that other influencing factors such as personality traits or hospital characteristics are important factors to consider.

## Conclusion

Our analyses indicate that individual obstetric interventions have a limited impact on the variability in the birth experience. However, women who underwent unplanned CS or AVD expressed significantly higher dissatisfaction with the childbirth experience compared to those who gave birth vaginally without any intervention. Further research is warranted to explore the substantial unexplained variance, particularly in the realm of the effects of empowerment and support during childbirth. Our study underscores the significance of professional support and efforts to alleviate fears during childbirth. Developing strategies with the obstetrical team may be beneficial to minimize the prevalence of adverse birth experiences during necessary birth control interventions.

### Electronic supplementary material

Below is the link to the electronic supplementary material.

Supplementary Material 1

## Data Availability

The datasets used and/or analysed during the current study are available from the corresponding author on reasonable request.

## Abbreviations

AVD: Assisted Vaginal Delivery
CS: Cesarean Section
CEQ: Childbirth Experience Questionnaire
CEQ2: Childbirth Experience Questionnaire 2
VE: Vacuum Extraction
