# Supplementary material for: Obstetric interventions’ effects on the birthing experience
Source: BMC Pregnancy Childbirth. 2024 Jul 27;24:508. doi: 10.1186/s12884-024-06626-5 (PMC11283698; doi:10.1186/s12884-024-06626-5)
Supplement: Supplementary file 1 — Supplementary Material 1 [file 12884_2024_6626_MOESM1_ESM.pdf]

Additional File 1

Table 3: CEQ-Total – Interventions

	Participation			Own capacity			Perceived safety			Professional support			Total		
**Characteristic**	**Beta**	**95% CI[1]**	**p-value**	**Beta**	**95% CI[1]**	**p-value**	**Beta**	**95% CI[1]**	**p-value**	**Beta**	**95% CI[1]**	**p-value**	**Beta**	**95% CI[1]**	**p-value**
Fundal Pressure	**-0.18**	-0.31, -0.05	<0.01	**-0.19**	-0.30, -0.07	<0.001	**-0.18**	-0.31, -0.06	<0.05	**-0.13**	-0.26, -0.01	<0.05	**-0.17**	-0.27, -0.07	<0.01
Episiotomy	-0.12	-0.28, 0.03	0.13	-0.07	-0.20, 0.07	0.3	-0.07	-0.22, 0.07	0.3	-0.08	-0.23, 0.06	0.3	-0.09	-0.21, 0.03	0.2
Instrumental Birth	**-0.30**	-0.50, -0.11	<0.01	**-0.32**	-0.49, -0.15	<0.001	**-0.37**	-0.56, -0.19	<0.001	**-0.27**	-0.46, -0.08	<0.01	**-0.32**	-0.47, -0.16	<0.001
Unplanned C-Section	-0.27	-0.59, 0.06	0.11	**-0.42**	-0.70, -0.15	<0.01	**-0.61**	-0.91, -0.31	<0.001	**-0.45**	-0.75, -0.14	<0.01	**-0.44**	-0.69, -0.19	<0.001
Age	0.00	-0.01, 0.01	0.9	-0.01	-0.02, 0.00	0.3	0.01	-0.01, 0.02	0.4	0.01	0.00, 0.02	0.2	0.00	-0.01, 0.01	0.7
Education															
Abitur	-0.06	-0.23, 0.10	0.4	0.00	-0.14, 0.14	>0.9	0.05	-0.11, 0.20	0.5	-0.11	-0.26, 0.05	0.2	-0.03	-0.16, 0.09	0.6
University	-0.11	-0.26, 0.04	0.2	-0.04	-0.17, 0.09	0.6	-0.02	-0.16, 0.12	0.7	**-0.17**	-0.32, -0.03	<0.05	-0.09	-0.20, 0.03	0.14
Parity (ref. nulliparous)	0.00	-0.11, 0.11	>0.9	**0.11**	0.02, 0.21	<0.05	0.01	-0.09, 0.11	0.9	-0.09	-0.19, 0.02	0.10	0.01	-0.08, 0.09	0.9
Birth Month (ref. may)	0.05	-0.05, 0.15	0.3	0.04	-0.04, 0.13	0.3	0.06	-0.03, 0.16	0.2	0.04	-0.05, 0.14	0.4	0.05	-0.03, 0.13	0.2
Birth Weight (ref. <3500g)	**0.13**	0.03, 0.23	<0.05	0.01	-0.08, 0.10	0.8	0.04	-0.05, 0.13	0.4	0.09	-0.01, 0.18	0.074	0.07	-0.01, 0.14	0.095
No. Obs.		733			733			733			733			733	
R^2		0.058			0.082			0.080			0.050			0.088	

[1]CI = Confidence Interval. Own calculations.
